# Solid-State Fermentation With *Aspergillus cristatus* Enhances the Protopanaxadiol- and Protopanaxatriol-Associated Skin Anti-aging Activity of *Panax notoginseng*

**DOI:** 10.3389/fmicb.2021.602135

**Published:** 2021-12-16

**Authors:** Sunmin Lee, Chagam Koteswara Reddy, Jeoung Jin Ryu, Seoyeon Kyung, Yonghwan Lim, Myeong Sam Park, Seunghyun Kang, Choong Hwan Lee

**Affiliations:** ^1^Department of Bioscience and Biotechnology, Konkuk University, Seoul, Seoul, South Korea; ^2^Resource Analysis Research Laboratory, Korea Ginseng Corporation, Daejeon, South Korea; ^3^COSMAX BTI R&I Center, Pangyo inno valley E, 255 Pangyo-ro, Bundang-gu, Seongnam-si, Gyeonggi-do, South Korea

**Keywords:** *Panax notoginseng*, *Aspergillus cristatus*, solid-state fermentation, mass spectrometry, protopanaxadiol, protopanaxatriol, skin anti-aging effect

## Abstract

A metabolomics approach was used to profile metabolites of *Panax notoginseng* fermented with *Aspergillus cristatus* in two ways, liquid-state fermentation (LF-P) and solid-state fermentation (SSF-P) and examine metabolite markers representing antioxidant activity and skin anti-aging. Protopanaxadiol (PPD) and protopanaxatriol (PPT) contents were higher in SSF-P than in LF-P and showed a multiplicative increase over the fermentation period of four days. PPD and PPT levels also correlated with antioxidant and anti-aging effects in skin, based on the mRNA expression of dermal extracellular matrix components. In the bioactivity validation assays, PPD and PPT significantly improved the expression of type-I collagen, fibrillin-1, and elastin in human dermal fibroblasts from both young and old subjects; these were comparable with the effects of the SSF-P extracts. Overall, our results suggest that changes in the metabolites of *P. notoginseng* fermented with *A. cristatus* enhance the quality and availability of bioactive compounds associated with skin anti-aging.

## Introduction

Ample research in recent years has been focused on microbiota-mediated biotransformation (Koppel et al., 2017). Immobilized edible fungi, such as *Aspergillus*, *Monascus*, *Rhizopus*, yeast, and *Mucor,* have also been utilized for biotransformation and efficient, natural bioactive metabolite production in industries (Zang et al., 2017). *Aspergillus cristatus* (synonym: *Eurotium cristatum*), termed as the “Golden Flowers Fungus” because of its yellow cleistothecium color, is a dominant fungal species utilized in the fermentation of Chinese brick tea (Ge et al., 2016; Ren et al., 2017). *A. cristatus* is an inoculum microbe for enhancing bioactivity and producing many beneficial metabolites because of its various enzymes, such as amylase, α-glucosidase, and β-glucosidase (Lee et al., 2021).

*Panax notoginseng* is a perennial herb belonging to the genus *Panax* anomaly Araliaceae that is widely used in traditional Chinese medicine. *P. notoginseng* contains a variety of chemical components including saponins, flavonoids, sterols, polysaccharides, amino acids, fatty acids, and various trace elements (Li et al., 2020). Specifically, the root is beneficial to human health due to its anti-atherosclerotic, antioxidant, anti-inflammatory, anti-hyperlipidemic, hypoglycemic, neuroprotective, anti-coagulation, and anti-tumor properties (Wang et al., 2016). Polysaccharides from *P. notoginseng* roots are strongly protective against oxidative stress and have been shown to extend lifespan in *Caenorhabditis elegans* (Feng et al., 2019).

*Panax* species has been studied using several technologies, including whole-genome sequencing, and high-performance liquid chromatography-mass spectrometry (Yang et al., 2016; Chen et al., 2017; Zhang et al., 2017). Among the different chemical compounds found in *P. notoginseng,* triterpenoid saponins, such as ginsenosides and notoginsenosides, are the major bioactive constituents that are actively utilized for clinical applications. Ginsenosides can be transformed into bioactive compounds, such as ginsenosides Rg3, Rg5, and RK1, by steaming and heating (Kim et al., 2018). Moreover, fermentation of ginsenosides in the presence of intestinal microflora results in the formation of metabolites, such as compound K, ginsenoside Rh1, and PPT (Kim, 2012). Microorganisms not only transform the chemical structure of triterpenoids, but also alter their biological activities. However, the influence of microflora-mediated biotransformation on the bioactivity potential of *P. notoginseng* is not well-understood and requires further studies.

Metabolomics has emerged as a useful and important tool in a variety of research areas, including food science, agriculture, and microbiology. Metabolite profiling can be used for the simultaneous monitoring of all metabolites in a sample to facilitate nutrient analysis and detect the changes in enzyme levels, as well as microbial activities (Lee et al., 2016). In this study, a metabolomics approach was used to evaluate the biochemical events underlying solid-state fermentation of *P. notoginseng* (SSF-P) and investigate the metabolite biomarkers representing the antioxidant activity and skin anti-aging property of *P. notoginseng*.

## Materials and Methods

### Chemicals and Reagents

Potato dextrose agar media (Difco) was purchased from Junsei Chemical Co., Ltd. (Tokyo, Japan). HPLC-grade water, acetonitrile, and methanol were obtained from Fisher Scientific Co., Ltd. (Pittsburgh, PA, United States). Analytical-grade sodium dihydrogen phosphate, sodium chloride, sodium hydroxide, sodium carbonate, disodium hydrogen phosphate, and diethylene glycol were purchased from Junsei Chemical Co., Ltd. (Tokyo, Japan). All remaining analytical-grade reagents and standard compounds used in this study were obtained from Sigma-Aldrich (St. Louis, MO, United States).

### Fungal Fermentation of *P. notoginseng*

The fungal culture used in this study (*Aspergillus cristatus* Cosmax-GF) was obtained from Cosmax BTI R&I center (Seongnam, Korea). Before fermentation, raw *P. notoginseng* roots (300 g) were washed with deionized water, soaked for 2 h (45% water content) at room temperature, and autoclaved at 121°C for 60 min. Fermentation of *P. notoginseng* was performed in two ways: solid-state fermentation (SSF-P) and liquid fermentation (LF-P). *A. cristatus* incubated on potato dextrose agar media at 30°C and collected by treated with 0.01% Tween-20 using Neubauer chamber. The final fungal spore was adjusted to approximately 2.0 × 10^5^ colony-forming units (CFUs)/ml. *A. cristatus* (2% v/w) was directly inoculated in *P. notoginseng* roots for solid-state fermentation. In case of liquid fermentation, we put *P. notoginseng* roots in the same amount of water and inoculated *A. cristatus* (2% v/w). After inoculation, the samples were incubated for 8 days at 30°C with 85% humidity. Samples were harvested every 48 h and immediately stored at −80°C until further analyses.

### Preparation of Samples

The moisture content of fermented *P. notoginseng* was reduced to less than 12% by drying at 50°C and then pulverized using a mortar and pestle. For sample extraction, each pulverized sample (200 mg) was mixed with aqueous ethanol (1.0 ml, 80%) and sonicated for 60 min in an ultrasonic water bath. After sonication, the sample dispersion was centrifuged at 2370 *g* for 10 min at 4°C, and the supernatant was filtered using a 0.22-μm Milex^®^ (Merck Millipore, Billerica, MA, United States). Then, the sample extracts were dried using a speed vacuum concentrator (Hanil, Seoul, Korea).

For gas chromatography time-of-flight mass spectrometry (GC-TOF-MS) analysis, each sample extract was subjected to two-staged derivatization. First, the oximation step was carried out by dissolving the sample extract with 50 μl of methoxyamine hydrochloride in pyridine (20 mg/ml) and incubated at 30°C for 90 min. Next, the silylation step was performed by adding 50 μl of N-Methyl-N-(trimethylsilyl) trifluoroacetamide (MSTFA) to the sample and the sample was incubated at 37°C for 30 min. Six replicates were analyzed for each sample.

For ultrahigh-performance liquid chromatography quadrupole orbitrap ion trap tandem mass spectrometry (UHPLC-Q-orbitrap-MS) analysis, each dried sample was dissolved in 80% ethanol and used. Six replicates were analyzed for each sample.

### Instrumentation

#### GC-TOF-MS Analysis

GC-TOF-MS analysis for *P. notoginseng* sample extracts was carried out using an Agilent 7890A GC system (Agilent Technologies, Santa Clara, CA, United States) coupled with the Pegasus HT TOF-MS (Leco Corporation, St. Joseph, MI, United States) and an Agilent 7,693 autosampler. The samples were separated on an Agilent Rtx-5MS column (30 m length × 0.25 mm i.d. × 0.25 μm film thickness; Restek Corp., Bellefonte, PA, United States), and the operational parameters were adapted from a study by Lee et al. (2020).

#### UHPLC-Q-orbitrap-MS/MS Analysis

UHPLC-Q-orbitrap-MS/MS analysis for *P. notoginseng* sample extracts was performed using a heated electrospray ionization source (Thermo Fischer Scientific Co., Ltd., CA, United States), equipped with a DIONEX UltiMate 3,000 UHPLC system (Ultimate 3,000 RS pump, Ultimate 3,000 RS column compartment, and Ultimate 3,000 RS autosampler; Dionex Corporation, CA, United States). Samples were separated on a hypersil gold C18 selectivity LC column (1.9 μm internal diameter, 50 mm × 2.1 mm; Thermo Fisher scientific Co., Ltd., CA, United States), and the operational parameters were adapted from a study by Lee et al. (2020).

### Data Processing and Multivariate Statistical Analysis

The raw data files from GC-TOF-MS and UHPLC-Q-orbitrap-MS/MS were converted into the computable document form (cdf) format using LECO Chroma TOF software v.4.44 (Leco Co., CA, United States) and Thermo Xcalibur v.2.2 (Thermo Fisher Scientific, CA, United States), respectively. After conversion, the converted data files were processed using the MetAlign software package^1^ to obtain a data matrix of accurate masses (*m/z*), normalized peak intensities, and retention times. Multivariate statistical analyses were performed using the SIMCA-P^+^ 12.0 software (Umetrics, Umea, Sweden), to analyze the differences among the metabolomics data of fermented *P. notoginseng* samples. Further, both unsupervised principal component analysis (PCA) and supervised partial least squares discriminant analysis (PLS-DA) were performed on the metabolomic data sets. The metabolites were identified by comparing their retention time and mass fragment patterns with standard compounds, in-house library data, and references.

### Bioactivity Assays

ABTS, DPPH, and FRAP assays were done to determine the *in vitro* antioxidant capacities of fermented *P. notoginseng* extracts (1 mg ml^−1^ methanol) by the method described by Lee et al. (2020). Trolox used as positive control in ABTS, DPPH, and FRAP assays. The results are presented as trolox equivalent antioxidant capacity (TEAC) concentration (mm) and as mean value of three analytical replicates.

#### ABTS Assay

The ABTS antioxidant assay was performed using a stock solution dissolving 7 mm ABTS in 2.45 mm potassium persulfate solution. For analysis, the ABTS solution was diluted in deionized water until an absorbance of 0.7 ± 0.02 (at 750 nm), measured using a spectrophotometer (Thermo Electron, Spectronic Genesys 6, Madison, WI, United States), was obtained. Each sample (20 μl) and diluted ABTS (180 μl) were added into a 96-well plate. The plate was incubated at room temperature for 6 min in the dark, and the absorbance of the samples was measured at 750 nm using a spectrophotometer.

#### DPPH Assay

For the DPPH assay, the fermented *P. notoginseng* extract (20 μl) was mixed with DPPH solution (180 μl, 0.2 mm in ethanol) in a 96 well plate, followed by incubation for 20 min at room temperature in the dark. The absorbance of the samples, which indicates the levels of DPPH free radicals, was measured at 515 nm using a spectrophotometer.

#### FRAP Assay

The FRAP assay was performed using a mixture of 10 mm 2,4,6-Tris (2-pyridyl)-s-triazine (TPTZ) solution in 40 mm HCl, 20 mm iron (III) chloride, and 300 mm acetate buffer at pH 3.6 (1:1:10, v:v:v). For the FRAP analysis, the fermented *P. notoginseng* extract (10 μl) was added to the FRAP reagent (300 μl), followed by incubation at room temperature for 6 min. The absorbance of the samples was measured at 570 nm using a spectrophotometer.

#### Total Phenol Content Assay

The total phenol content (TPC) assay of fermented *P. notoginseng* extracts was performed *via* two steps. First, 20 μl of fermented *P. notoginseng* extract in 80% methanol (1 mg ml^−1^) was added to 100 μl of 0.2 N Folin-Ciocalteu’s phenol reagent, followed by incubation for 5 min at room temperature in the dark. In the next step, 80 μl of 7.5% Na_2_CO_3_ was added to the samples, and the resulting reaction mixtures were incubated for 60 min. Finally, the absorbance of the samples was measured at 750 nm using a spectrophotometer. The assay results were expressed in terms of the gallic acid equivalent of the activity (μg ml^−1^) and as the mean value of three analytical replicates.

### Cell Cultures

Primary human dermal fibroblast (HDFs) from young (20 y) and old (over 65 y) subjects were purchased from PromoCell (Heidelberg, Germany). The related culture media and DetachKit were also obtained from PromoCell. The HDFs were grown in specific fibroblast medium (Fibroblast Growth Medium 2, PromoCell, Cat no. C-23020) enriched with Supplement Mix/Fibroblast Growth Medium 2 (PromoCell, Cat no. C-39325) and 1% Penicillin-Streptomycin (PS) at 37°C in a 5% CO_2_ incubator. When cultured HDFs reached approximately 80% confluence, they were subsequently cultured or seeded into the appropriate wells for the different treatments.

### Real-Time Polymerase Chain Reaction

Total RNA from cell pellets was isolated using TRIzol reagent and quantified using a spectrophotometer. The cDNA was synthesized in a total reaction volume of 20 μl; the reaction mixture contained 2 μg of total RNA, oligo (dT), and reverse transcription premix under the following reaction conditions: 45°C for 45 min, followed by 95°C for 5 min. Gene expression was quantified using RT-PCR, and the resultant data were analyzed using the StepOne Plus™ system software (Applied Biosystems, Foster City, CA, United States). RT-PCR amplifications were performed using SYBR Green PCR Master Mix with premixed ROX (Applied Biosystems, Foster City, CA, United States) and primers (Bioneer, Daejeon, Korea) in an ABI 7300 instrument according to the manufacturer’s protocol. The reaction conditions were as follows: initiation at 95°C for 10 min, followed by cycling conditions of 95°C for 15 s, 60°C for 30 s, and 72°C for 30 s for 40 cycles. Beta-actin was used as an internal control.

## Results

### Comparison of the Metabolomes and Bioactivities of *P. notoginseng* Following Liquid Fermentation and Solid-State Fermentation

**Figure 1:**
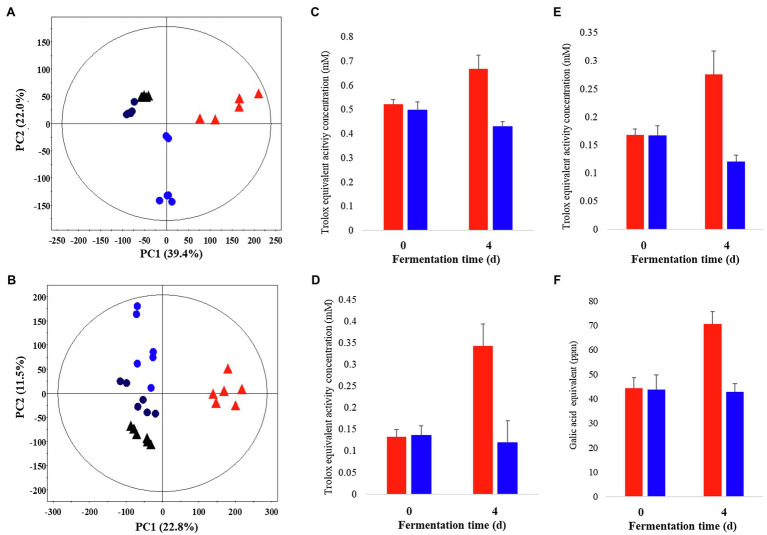
Principal component analysis (PCA) score plot derived from GC-TOF-MS **(A)** and UHPLC-Q-orbitrap-MS **(B)** data sets. Comparison of the bioactivities of *P. notoginseng* during SSF-P and LF-P **(C)** ABTS, **(D)** DPPH, **(E)** FRAP, **(F)** Total phenol contents. 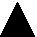, black: day 0 of solid-state fermentation (SSF-P); 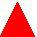, red: day 4 of SSF-P; 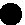, black: day 0 of liquid fermentation (LF-P); 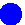, blue: day 4 of liquid fermentation (LF-P).

Metabolite profiling was performed on *P. notoginseng* following LF-P and SSF-P, using GC-TOF-MS and UHPLC-Q-orbitrap-MS combined with multivariate analysis, to determine the metabolites associated with the PCA score plots based on the GC-TOF-MS (Figure 1A) and UHPLC-Q-orbitrap-MS (Figure 1B; Supplementary Figure 1) data sets showed that the different types of fermentation process lead to changes in metabolite distributions of *P. notoginseng*. Among LF-P and SSF-P, the PCA score plots based on GC-TOF-MS and UHPLC-Q-orbitrap-MS analysis were accounted for 61.4% of the total variability (PC1, 39.4%; PC2, 22.0%) and 34.3% of total variability (PC1, 22.8% PC2, 11.5%), respectively. In the next step, considering the differential impacts of the fermentation environment on *P. notoginseng* metabolite distributions, we examined the bioactivities associated with each fermentation procedure. Our results, based on the ABTS, DPPH, FRAP, and TPC assays, showed that the bioactivities following SSF-P were higher than those in case of LF-P on the fourth day of fermentation (Figures 1C–F).

### Temporal Metabolomes and Bioactivities of *P. notoginseng* Following Solid-State Fermentation

#### Temporal Metabolomes of *P. notoginseng* Following Solid-State Fermentation

**Figure 2:**
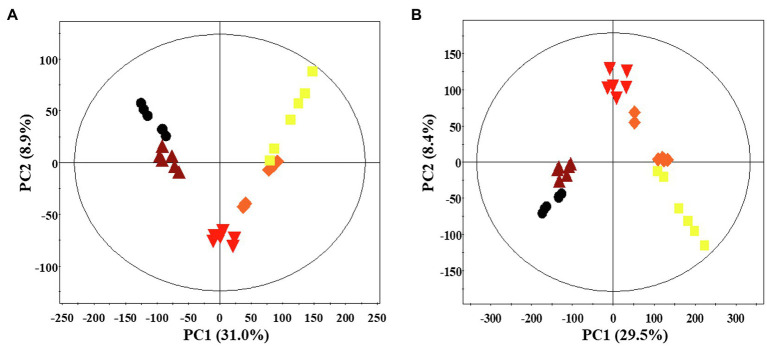
Principal component analysis (PCA) score plot of *P. notoginseng* during SSF-P derived from the GC-TOF-MS **(A)** and UHPLC-Q-orbitrap-MS **(B)** data sets. 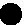, day 0; 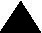, 2 d; 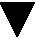, 4 d; 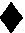, 6 d; 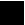, 8 d.

A multivariate statistical analysis was carried out using the GC-TOF-MS and UHPLC-Q-orbitrap-MS data sets to investigate the temporal metabolomes following SSF-P (Figure 2). The PCA score plots derived from the GC-TOF-MS (Figure 2A) and UHPLC-Q-orbitrap-MS (Figure 2B), data sets for the extracts of *P. notoginseng* fermented under different fermentation periods exhibited a clustered pattern. The PCA score plot obtained from GC-TOF-MS analysis represented a total variability of 39.9% (PC1, 31.0%; PC2, 8.9%; Figure 2A), whereas the PCA score plots based on the UHPLC-Q-orbitrap-MS data sets (Figure 2B) represented a total variability of 37.9% (PC1, 29.5%; PC2, 8.4%). PLS-DA models with variable importance in projection values (VIP > 0.7 values and *value of p* <0.05) were derived from the GC-TOF-MS and UHPLC-Q-orbitrap-MS data sets, respectively. A total of 36 significant discriminant metabolites, including 22 primary and 14 secondary metabolites, were selected for fermented *P. notoginseng* on the basis of the PLS1 and PLS2 components. Overall, three amino acids (GABA, glutamic acid, and pyroglutamic acid), three fatty acids (linoleic acid, oleic acid, and stearic acid), six organic acids (citric acid, fumaric acid, malonic acid, malic acid, gluconic acid, and succinic acid), ten sugar and sugar alcohols (adonitol, erythritol, glycerol, glucuronic acid, glyceryl-glycoside, myo-inositol, ribose, sorbitol, sucrose, and xylose), and 14 triterpenoids (ginsenoside-Rb1, Rb2, Rc, Rd., Rf, Rg2, Rg3, Rh1, glucoginsenoside-Rf, notoginsenoside-R1, -R2, and -R4, protopanaxadiol (PPD), and PPT) were putatively identified. The peak area of identified metabolites was represented by the fold change obtained following normalization to the average fermentation time for each metabolite. We observed a common trend, dependent on the fermentation time, among the metabolic data sets of the extracts of *P. notoginseng* fermented using the two different methods.

Table 1 shows the relative distributions of primary and secondary metabolites in *P. notoginseng*. Specifically, following SSF-P, the levels of many fatty acids (linoleic acid, oleic acid, and stearic acid) showed an increasing trend over the fermentation period. The contents of most sugars and sugar alcohols (adonitol, erythritol, glycerol, glucuronic acid, glyceryl-glycoside, ribose, sorbitol, and xylose) also increased significantly, whereas sucrose levels reduced significantly. Among the selected secondary metabolites, the levels of ginsenosides, including ginsenoside-Rb1, Rb2, Rc, Rd., Rf, ginsenoside-Rg2, Rh1, and notoginsenoside-R1, -R2, and -R4 showed a decreasing trend, whereas the levels of ginsenoside-Rg3 and glucoginsenoside-Rf showed an increasing trend. Finally, the levels of PPD and PPT significantly increased during the fermentation period.

**Table 1:** Relative distributions of metabolites analyzed by GC-TOF-MS and UHPLC-Q-orbitrap-MS in SSF-P (solid-state fermentation *P. notoginseng*) fermented with *A. cristatus.*

	Fermentation time (d)				
	0	2	4	6	8
**Amino acids**					
GABA	1.19	1.19	0.97	0.92	0.74
Glutamic acid	0.71	0.88	1.15	1.23	1.02
Pyroglutamic acid	1.05	1.09	0.99	1.01	0.86
**Fatty acids**					
Linoleic acid	0.92	1.00	1.02	1.04	1.01
Oleic acid	0.81	0.91	0.99	1.15	1.14
Stearic acid	0.96	1.01	0.96	1.03	1.03
**Organic acids**					
Citric acid	1.04	1.05	1.05	1.01	0.85
Fumaric acid	0.81	0.84	1.05	1.17	1.13
Malonic acid	1.06	1.07	1.02	1.01	0.84
Malic acid	0.99	1.00	1.01	1.02	0.98
Gluconic acid	1.07	1.04	1.00	0.96	0.94
Succinic acid	0.93	0.97	1.08	1.10	0.92
**Sugar & sugar alcohols**					
Adonitol	0.93	0.93	1.02	1.00	1.12
Erythritol	0.66	0.75	1.11	1.20	1.29
Glycerol	0.83	0.97	1.04	1.07	1.08
Glucuronic acid	0.80	0.84	1.09	1.12	1.16
Glyceryl-glycoside	0.74	0.82	0.99	1.15	1.30
Myo-inositol	1.02	1.03	0.99	0.98	0.97
Ribose	0.78	0.92	1.12	1.09	1.08
Sorbitol	0.65	0.77	1.17	1.20	1.22
Sucrose	1.17	1.16	1.01	0.90	0.76
Xylose	0.80	0.82	1.04	1.13	1.22
**Terpenoids**					
Ginsenoside-Rb1	1.08	1.05	1.02	0.90	0.94
Ginsenoside-Rb2	1.32	1.09	1.07	0.89	0.63
Ginsenoside-Rc	1.41	1.05	0.96	0.82	0.76
Ginsenoside-Rd	1.12	1.02	1.03	0.90	0.93
Ginsenoside-Rf	1.21	1.10	1.03	0.86	0.80
Ginsenoside Rg2	1.15	0.99	1.08	0.91	0.87
Ginsenoside Rg3	0.81	0.53	0.89	1.60	1.17
Ginsenoside- Rh1	1.20	1.06	1.03	0.83	0.88
Glucoginsenoside-Rf	0.86	0.76	0.91	1.12	1.36
Notoginsenoside-R1	1.05	1.05	0.99	1.00	0.91
Notoginsenoside-R2	0.96	1.13	1.05	0.96	0.90
Notoginsenoside-R4	1.37	1.15	0.90	0.91	0.68
Protopanaxadiol	0.05	0.05	1.61	1.64	1.65
Protopanaxatriol	0.00	1.24	1.32	1.18	1.25

*The values of the table for metabolite levels represent their average fold change values*.

#### Antioxidant Activities of *P. notoginseng* Following Solid-State Fermentation and Effects of Extracts Obtained Following SSF-P on Skin Aging

**Figure 3:**
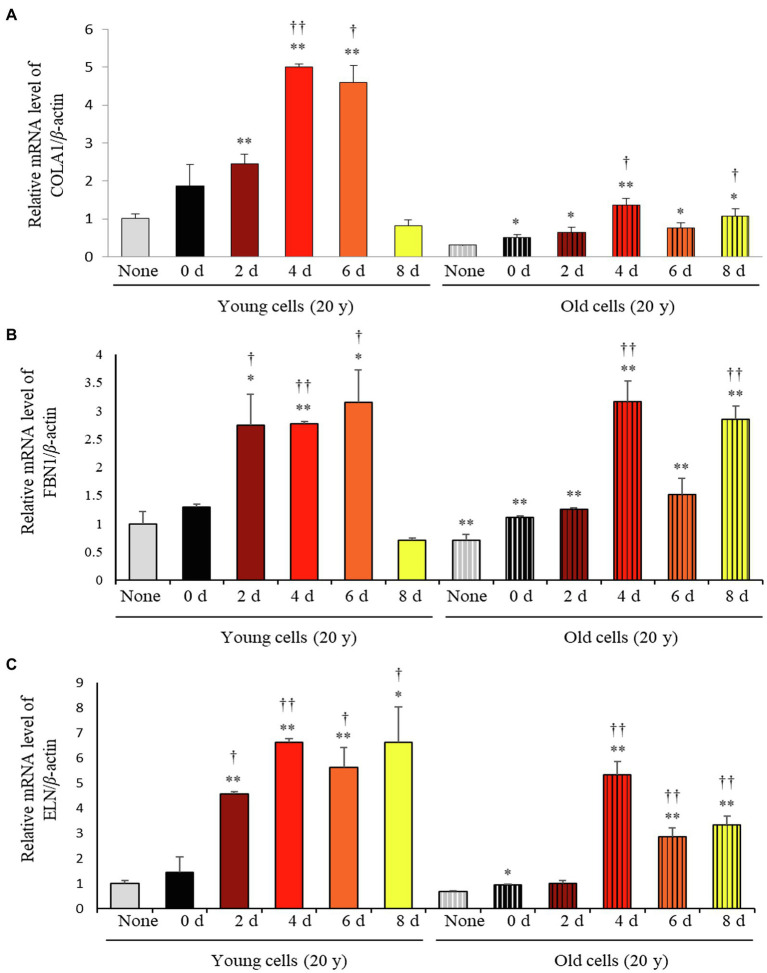
Relative mRNA expression levels of **(A)** COLA1, **(B)** FBN1, and **(C)** ELN in primary normal human dermal fibroblasts (HDFS) incubated with *P. notoginseng* during SSF fermentation. ^*^p < 0.05, ^**^p < 0.01 vs. None (Young & old), ^†^*p* < 0.05, ^††^*p* < 0.0 vs. SSF-P day (young & old).

Considering the varying impact of the solid-state fermentation time on *P. notoginseng* metabolites, we analyzed antioxidant activities over the fermentation period using the DPPH, ABTS, FRAP, and TPC assays. All these assays revealed that the bioactivities increased significantly with increasing fermentation time (Supplementary Figure 2). The effects of fermented *P. notoginseng* on skin aging were investigated using HDFs from subjects belonging to two age groups, the twenties (young) and sixties (old), for investigating the intrinsic aging states of the dermis. The effects of SSF-P extracts on skin aging-associated factors, such as type-I collagen (COLA1), fibrillin-1 (FBN1), and elastin (ELN), were monitored. As shown in Figure 3, SSF-P extracts significantly increased the expression of COLA1, FBN1, and ELN in HDFs obtained from both young and old subjects.

### Correlation Between Bioactivities and Significant Discriminant Metabolites

The bioactivity of *P. notoginseng* SSF-P extracts is mainly due to its distinct metabolite composition. The spatial distributions of these compounds among different phylogenic groups and plant components are remarkably discriminant.

**Figure 4:**
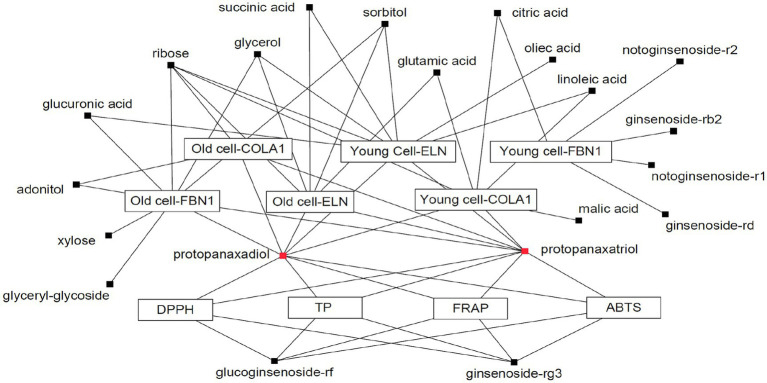
Correlation network between metabolites and bioassays. Box: metabolites; Eclipse: bioassay.

A Pearson correlation analysis tentatively identified compounds that maximally contributed to the bioactivities of the extracts obtained following SSF-P using *A. cristatus*. The correlation network was evaluated for variables with positive Pearson correlation values (Figure 4). Citric acid, glutamic acid, linoleic acid, malic acid, PPD, PPT, ribose, and succinic acid were correlated with COLA1 levels in HDFs from young subjects, while adonitol, PPD, PPT, and ribose correlated with COLA1 levels in HDFs from old subjects. Similarly, citric acid, ginsenoside-Rb2, ginsenoside-Rd, notoginsenoside-R1, and notoginsenoside-R2 correlated with FBN1 levels in HDFs from young subjects, whereas adonitol, glucuronic acid, glycerol, glyceryl-glycoside, PPD, PPT, ribose, sorbitol, and xylose correlated with FBN1 levels in HDFs from old subjects. Finally, glucuronic acid, glycerol, linoleic acid, oleic acid, PPD, PPT, ribose, succinic acid, and sorbitol correlated with ELN levels in HDFs from young subjects, while glutamic acid, glycerol, PPD, PPT, ribose, sorbitol, and succinic acid correlated with ELN levels in HDFs from old subjects. PPD, PPT, glucoginsenoside-Rf, and ginsenoside-Rg3 showed positive correlations with the bioactivities measured using the ABTS, DPPH, FRAP, and TPC analyses. Intriguingly, PPD and PPT expressed strong positive correlations with bioactivity measured in all the assays performed and cell-based assays aimed at evaluating skin aging.

### Bioactivity Validation of Proposed Metabolites

**Figure 5:**
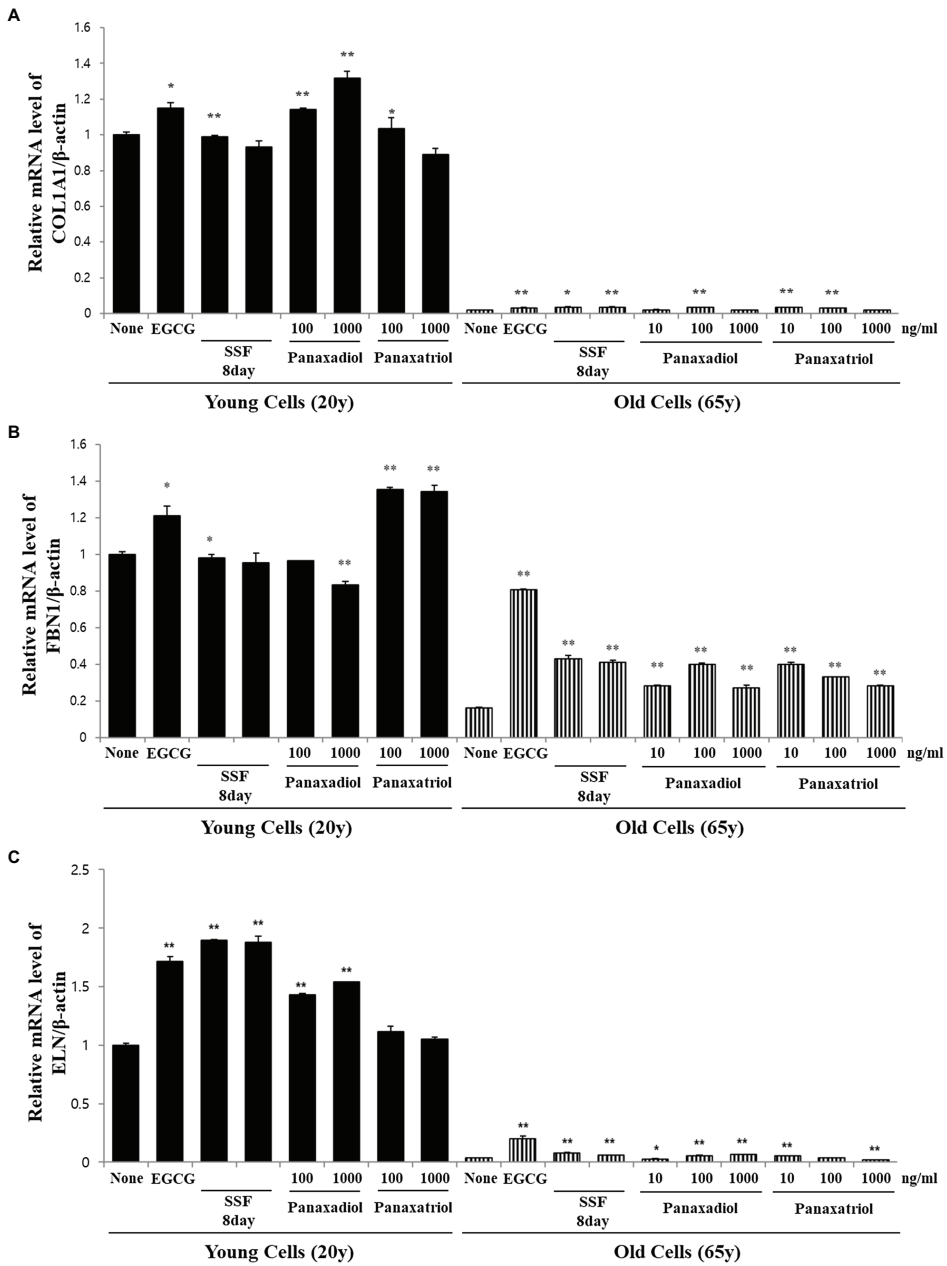
Relative mRNA expression levels of **(A)** COLA1, **(B)** FBN1, and **(C)** ELN in primary normal HDFS treated with protopanaxadiol and protopanaxatriol. ^*^p < 0.05, ^**^p < 0.01 vs. None.

Based on the results of metabolite profiling and correlation analysis, we hypothesized that the two metabolites PPD and PPT are bioactive components with strong skin anti-aging activity. To confirm the proposed hypothesis, we performed bioactivity validation assays using commercial standards for PPD, PPT, and epigallocatechin gallate (EGCG; used as positive control; Figure 5). The EC50 values of standard compounds could not be determined for some bioactivity assays (ABTS, DPPH, and FRAP) due to aggregation of reaction mixture. However, the results of the bioactivity validation analysis showed that both PPD and PPT significantly improved the expression of COLA1, FBN1, and ELN in HDFs from both young and old subjects; these findings were comparable with the effects observed for EGCG and *P. notoginseng* extracts obtained on the 8^th^ day of SSF-P.

## Discussion

In this study, untargeted metabolomics was used for evaluating the fermentation of *P. notoginseng* roots with *A. cristatus*. The chemical composition of *P. notoginseng* roots varied significantly over the fermentation period, possibly due to several factors, including the differences in the quantity or activity of enzymes released from *A. cristatus* resulting from the increasing fermentation time or the fermentation procedure. Previously, Son et al. (2018) reported differences in the metabolite levels and bioactivities between solid-state fermentation and submerged fermentation with *Aspergillus oryzae.* In this study, we primarily focused on solid-state fermentation, as higher bioactivity was observed following SSF-P than that following LF-P, along with a distinctive pattern shown by the samples in the PCA score plots. Additionally, numerous studies in the recent years have focused on solid-state fermentation and its applications, including production of enzymes, biomolecules, phenolics, organic compounds, and aromas for food fermentation, pharmaceutical, and cosmetic industries (Soccol et al., 2017).

The multivariate analysis of SSF-P data derived from GC-TOF-MS and UHPLC-Q-orbitrap-MS exhibited a central tendency with increased bioactivities based on the period of fermentation. The levels of PPD-type ginsenosides (Rb1, Rb2, Rc, and Rd), PPT-type ginsenosides (notoginsenoside R1) decreased, whereas the levels of PPD and PPT increased with increasing fermentation period. Using microbial enzymes for the conversion of major ginsenosides in *P. notoginseng* to bioactive components is valuable in the development of ginseng-based products (Wang et al., 2011; Zang et al., 2017). Liu et al. (2010) described that *Aspergillus niger* can convert ginsenoside-Rf into PPT by glycosidase, and the *β*-glucosidase secreted from *Aspergillus* plays a key role in the hydrolysis of biomass (Sørensen et al., 2011). We have also confirmed the *β*-glucosidase activity of *A. cristatus* using an enzyme kit (data not shown).

Antioxidant activities, as evaluated by the ABTS, DPPH, FRAP, and TPC assays, as well as mRNA expression levels of genes, such as COLA1, FBN1, and ELN, continuously increased over the period of fermentation. Several plants contain antioxidant compounds, which are involved in the scavenging of free radicals and eliminating the byproducts of metabolism to overcome the typical effects of aging (Mukherjee et al., 2011). It is reported that American ginseng root and berry, as well as Korean ginseng leaf extract, possess antioxidant properties (Wang et al., 2009). Thus, the increase in the levels of antioxidant compounds observed following the SSF-P in this study could prove to be useful in enhancing the nutritional and functional value of *P. notoginseng*. To explore the protective effects of SSF-P on skin aging, the expression levels of aging biomarkers in HDFs exposed to SSF-P extracts were measured by semi-quantitative PCR. Wrinkles, a major feature of skin aging, are primarily a result of molecular modifications of the dermis (Tang et al., 2017), and the extracellular matrix, which consists of collagen, elastin fibers, and fibrillin, is majorly involved in the process of cutaneous aging (Jung et al., 1997).

Correlation studies and validation assays were performed to identify the bioactivity and efficacy of the identified biomarkers during the fermentation period (Figure 4). Among the 36 discriminant metabolites, four saponins, including glucoginsenoside-Rf, ginsenoside Rg3, PPD, and PPT, were positively correlated with antioxidant activity. Ginsenosides represent one of the major bioactive components in white ginseng; they possess powerful antioxidant effects and high bioactivities (Lim et al., 2010). Of the saponins, six metabolites, including notoginsenoside-R2, ginsenoside-Rb2, notoginsenoside-R1, ginsenoside-Rd, PPT, and PPD, were correlated with skin aging effects. Previous studies have suggested that red ginseng extracts have beneficial effects on photoaging and reduce facial wrinkles by improving type-I procollagen gene and protein expression (Cho et al., 2009). Ginsenoside-Rb1, a metabolite of *P. notoginseng*, has anti-aging and hydrating effects and is used in cosmetic products for skin protection (Kim et al., 2018). In this study, we observed that PPD and PPT were positively correlated with both antioxidant activity (measured *via* the ABTS, DPPH, FRAP, and TPC analyses), as well as skin anti-aging effects (based on the increased mRNA expression of COLA1, ELN, and FBN1). Overall, our study identified biomarkers correlated with the antioxidant and anti-aging effects of *P. notoginseng* extracts obtained following SSF-P using *A. cristatus*, based on metabolic bioconversions and the resulting metabolite levels. In the present study, metabolite profiling and integrated bioassays were used to evaluate the biochemical activities characterizing the SSF-P with *A. cristatus* and identify potential metabolite biomarkers for antioxidant activity and skin anti-aging effects. The antioxidant activity and skin anti-aging effects following SSF-P were observed to increase over the fermentation period. In conclusion, we propose protopanaxadiol and protopanaxatriol as biomarkers for bioassays to study *P. notoginseng* fermentation with *A. cristatus*. Thus, our results provide a direction for the efficient fermentation of *P. notoginseng* with *A. cristatus* aimed at the production of bioactive compounds.

## Data Availability Statement

The original contributions presented in the study are included in the article/Supplementary Material, and further inquiries can be directed to the corresponding author.

## Author Contributions

CL: conceptualization and supervision. SL: methodology, software, and writing – original draft preparation. CR: validation. JR: investigation. JR, YL, MP, and SK: resources. SK: data curation and project administration. CR and CL: writing – review and editing. SL and SK: visualization. All authors have read and agreed to the published version of the manuscript.

## Funding

This study was supported by Traditional Culture Convergence Research Program through the National Research Foundation of Korea (NRF) funded by the Ministry of Science, ICT & Future Planning (NRF-2017M3C1B5019303) and the Basic Research Lab Program (Grant no. 2020R1A4A1018648), through the National Research Foundation grant funded by the Ministry of Science and ICT, Republic of Korea.

## Conflict of Interest

The authors declare that the research was conducted in the absence of any commercial or financial relationships that could be construed as a potential conflict of interest.

## Publisher’s Note

All claims expressed in this article are solely those of the authors and do not necessarily represent those of their affiliated organizations, or those of the publisher, the editors and the reviewers. Any product that may be evaluated in this article, or claim that may be made by its manufacturer, is not guaranteed or endorsed by the publisher.

## Footnotes

^1^ http://www.metalign.nl

## Supplementary Material

The Supplementary Material for this article can be found online at:

https://www.frontiersin.org/articles/10.3389/fmicb.2021.602135/full#supplementary-material
