# Supplementary material for: Solid-State Fermentation With Aspergillus cristatus Enhances the Protopanaxadiol- and Protopanaxatriol-Associated Skin Anti-aging Activity of Panax notoginseng
Source: Front Microbiol. 2021 Dec 16;12:602135. doi: 10.3389/fmicb.2021.602135 (PMC8718098; doi:10.3389/fmicb.2021.602135)

Supplementary Material

Supplementary Figure 1. UHPLC-Q-orbitrap-MS chromatogram in LF-P (A) and SSF-P (B) fermented with *A. cristatus.* (1) glucoginsenoside rf, (2) notoginsenoside r1, (3) ginsenoside Rg1, (4) notoginsenoside r4, (5) ginsenoside rb1, (6) notoginsenoside r2, (7) ginsenoside rb2, (8) ginsenoside rh1, (9) ginsenoside rd, (10) ginsenoside rc, (11) ginsenoside rg2, (12) protopanaxatriol, (13) ginsenoside rg3, (14) protopanaxadiol

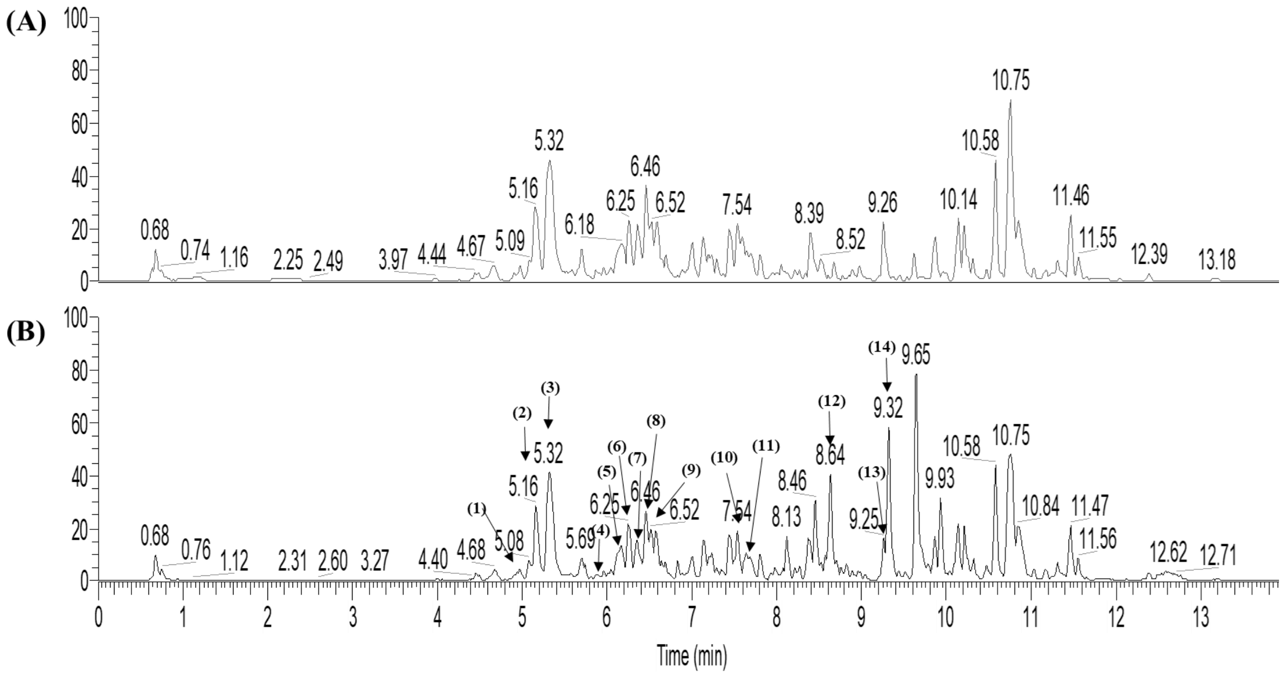

Supplementary Figure 2. Comparison of the bioactivities of *P. notoginseng* during SSF-P. (A) ABTS radical-scavenging activity, (B) DPPH radical-scavenging activity, (C) FRAP assay, (D) Total phenol contents

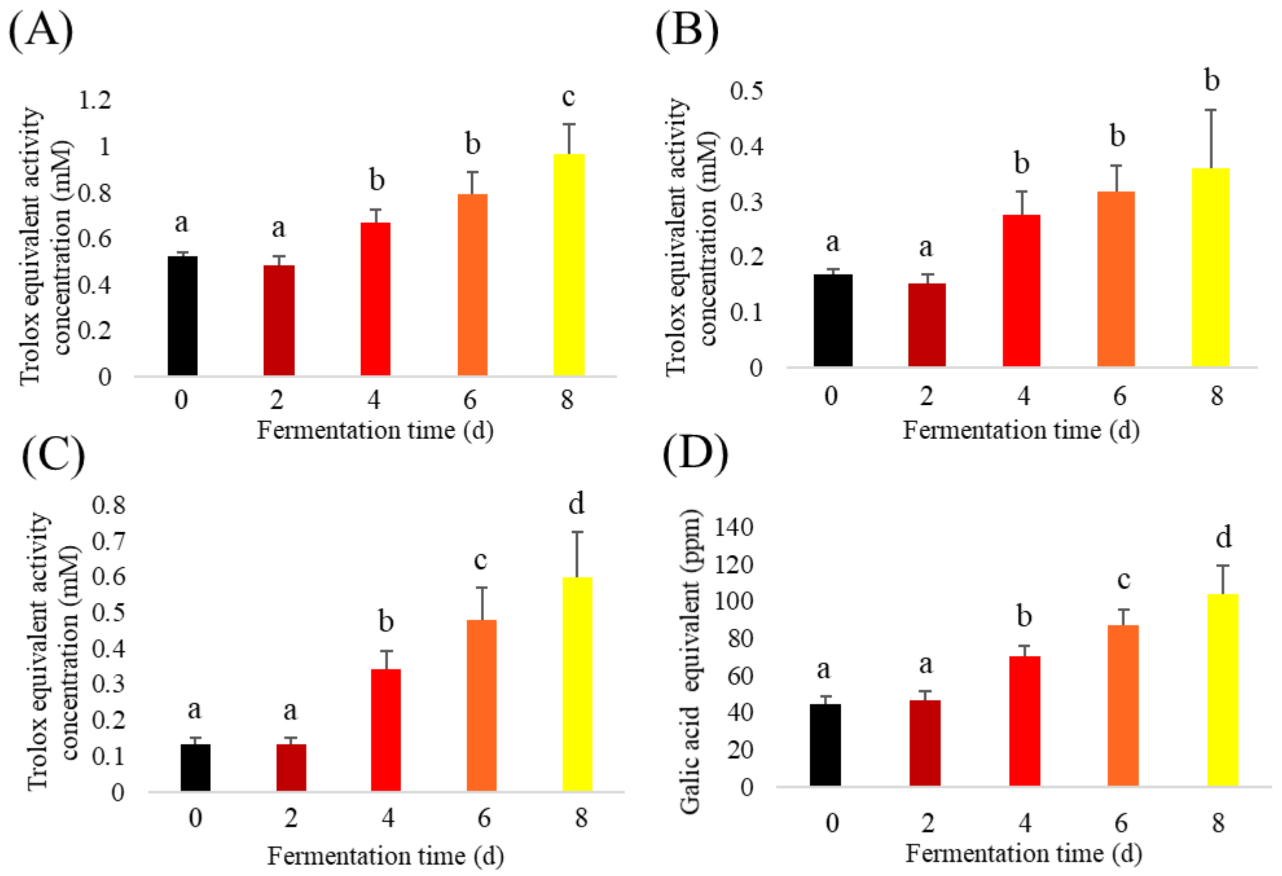

Supplement: Supplementary file 1 [file Data_Sheet_1.pdf]
